# Systematic review of the health-related quality of life issues facing adolescents and young adults with cancer

**DOI:** 10.1007/s11136-017-1520-x

**Published:** 2017-03-01

**Authors:** Samantha C. Sodergren, Olga Husson, Jessica Robinson, Gudrun E. Rohde, Iwona M. Tomaszewska, Bella Vivat, Rebecca Dyar, Anne-Sophie Darlington, On behalf of the EORTC Quality of Life Group

**Affiliations:** 1 Faculty of Health Sciences, University of Southampton, Southampton, UK; 2 Department of Medical Psychology, Radboud University Medical Center, Nijmegen, The Netherlands; 3 Faculty of Health and Sport Sciences, University of Agder, Kristiansand, Norway; 4 Department of Medical Education, Jagiellonian University Medical College, Kraków, Poland; 5 Marie Curie Palliative Care Research Department and Division of Psychiatry, University College London, London, UK; 6 Torbay Hospital, Torquay, UK; 7 Department of Clinical Research, Sorlandet Hospital, Kristiansand, Norway

**Keywords:** Adolescents and young adults (AYAs), Health-related quality of life (HRQoL), Cancer, Patient reported outcome measures (PROMs)

## Abstract

**Purpose:**

For adolescents and young adults (AYAs), the impact of a cancer diagnosis and subsequent treatment is likely to be distinct from other age groups given the unique and complex psychosocial challenges of this developmental phase. In this review of the literature, we report the health-related quality of life (HRQoL) issues experienced by AYAs diagnosed with cancer and undergoing treatment.

**Methods:**

MEDLINE, EMBASE, CINAHL, PsychINFO and the Cochrane Library Databases were searched for publications reporting HRQoL of AYAs. Issues generated from interviews with AYAs or from responses to patient reported outcome measures (PROMs) were extracted.

**Results:**

166 papers were reviewed in full and comprised 72 papers covering 69 primary studies, 49 measurement development or evaluation papers and 45 reviews. Of the 69 studies reviewed, 11 (16%) used interviews to elicit AYAs’ descriptions of HRQoL issues. The majority of the PROMs used in the studies represent adaptations of paediatric or adult measures. HRQoL issues were organised into the following categories: physical, cognitive, restricted activities, relationships with others, fertility, emotions, body image and spirituality/outlook on life.

**Conclusion:**

The HRQoL issues presented within this review are likely to be informative to health care professionals and AYAs. The extensive list of issues suggests that the impact of a cancer diagnosis and treatment during adolescence and young adulthood is widespread and reflects the complexities of this developmental phase.

## Introduction

### Background

In recent years, there has been increased emphasis in health care on the development and use of measures which give patients the opportunity to rate the physical and psychosocial impact of illness and treatment [1]. These patient reported outcome measures (PROMs) are informative for both patient and clinician and contribute valuable information for clinical trials and medical decision making [2].

For one group of patients, adolescents and young adults (AYA) with cancer, health-related quality of life (HRQoL) assessment is especially relevant as, compared with children and older adults, this group is regarded as particularly vulnerable [3, 4]. Adolescence marks the transitional stage linking childhood and adulthood in which puberty occurs. The World Health Organization [5] defines adolescents as 10–19 year olds although it is recognised that this age definition is not fixed and varies according to gender, biological, cultural and socio-economic factors. There is also fluidity in what defines a young adult and while the World Health Organization’s definition of young people includes 10–24 year olds, the age range used by other organisations, such the Adolescent and Young Adult Oncology Progress Review Group, extends to 39 year olds [6].

There are several issues that warrant the classification of AYAs as different from paediatrics or older adults with cancer. Firstly, the epidemiology of cancer in AYAs differs from other age groups. While cancer in AYAs is relatively rare, its incidence is increasing and is higher than that in children [7–11]. In the UK, 2000 AYAs (aged between 15 and 24 years) are diagnosed with cancer each year which is the second cause of death in this age group [12]. Cancer types in this group are less prevalent in other age groups and there is evidence to suggest that survival outcomes for some cancers in this group have not improved in line with figures achieved for paediatric or older adult groups [13–15]. Ten per cent of tumours seen in AYAs are predominantly childhood tumours, while 30% of tumours have a peak in adolescence and include Hodgkin lymphoma, Ewing’s sarcoma, osteosarcoma, germ-cell tumours and rare soft-tissue sarcomas. A final 60% are early-onset adult cancers [14, 15].

Adolescence and early adulthood is a unique and complex developmental phase characterised not only by significant physical and cognitive changes but also critical psychosocial challenges, relating to self-identity, peer relationships, development of autonomy, and sexuality. This also represents an important life stage with regard to education and future goal setting. Given the unique life circumstances and challenges of this group, it could be argued that the experience and impact of cancer on AYAs’ HRQoL will be distinct from other age groups.

According to the World Health Organization Quality of Life Group, HRQoL can be defined as a multi-dimensional construct shaped by physical health, psychological state, level of independence, social relationships, personal beliefs and their relationship to important environmental features with HRQoL appraisals shaped by coping strategies, goals and expectations [16]. Thus, it follows that HRQoL measures for this age group should be tailored to AYA-specific issues. HRQoL measures developed for paediatric practice often incorporate the adolescent years as their recommended upper age limit for use. In addition, adolescence and early adulthood are often included within the lower age limits of adult measures.

### Objectives

This review focuses on HRQoL issues experienced by AYAs during their diagnosis and treatment for cancer and refers to descriptive accounts provided during interviews with AYAs with cancer and the content of AYA-specific or adapted PROMs. This review will also pave the way for a discussion on how these HRQoL issues might be distinct from those important to children and older adults. To the best of our knowledge, this review is the first of its kind in terms of systematically reviewing the literature for HRQoL issues faced by AYAs with the specific focus on the diagnosis and treatment period rather than the post-treatment or survivorship phase which presents its own challenges, such as living with long lasting effects of treatment, anxiety over leaving the hospital system, readjusting to life after treatment and fear of recurrence [17].

## Methods

The protocol for this systematic review was informed by the Centre for Reviews and Dissemination guidance for undertaking reviews in health care [18] and the reporting follows the preferred reporting items of systematic reviews and meta-analyses (PRISMA) guidelines [19]. The protocol is available from the first author.

### Search strategies and criteria for considering studies

An initial scoping of the literature using MEDLINE with the following search terms and their synonyms “cancer”, “adolescent”, “young adult” and “health-related quality of life” generated 8635 records. A revised, more focused strategy was adopted and verified by two medical librarians. This strategy used exact major headings (MM) as well as medical subject headings (MeSH terms) and applied the focus/major concept options with Boolean logic rules (Table 1). MEDLINE, EMBASE, CINAHL, PsychINFO and the Cochrane Library Databases were searched for publications up until May 2015 with no defined start date.

**Table 1:** Revised strategy

Step	Search term
1	MM^b^ Health status indicators (MeSH^c^, Major Concept)
2	MM Patient outcome assessment (MeSH, Major Concept)^a^
3	MM Outcome assessment (Health care) (MeSH, Major Concept)^a^
4	MM Quality of life (MeSH, Major Concept)
5	1 or 2 or 3 or 4
6	Young adult (MeSH)
7	Adolescent
8	6 or 7
9	MM Neoplasms+ (MeSH, Major Concept, Exploded)
10	5 and 8 and 9

^a^Terms not identified in PsychINFO, EMBASE or CINAHL

^b^Exact major subheading search

^c^Medical subheading

The first phase of the selection process involved three independent reviewers (S.S., O.H., J.R.) identifying eligible papers based on their titles and abstracts. S.S. screened all the papers while O.H. and J.R. independently reviewed half the records each. Papers selected by either reviewer were included in the second phase of the review process. The process was monitored by a further reviewer (A-S.D.). English language publications were eligible for inclusion if they assessed HRQoL in AYAs with cancer from the perspective of the AYA rather than proxy, although papers including parent assessments alongside AYAs self-reports were accepted. Papers describing trials or patient cohort studies were included. Reviews and reports were also considered for descriptive and cross-referencing purposes but not for data extraction to avoid duplication. Papers describing the development of measures to assess HRQOL in AYAs with cancer were also eligible for inclusion for descriptive purposes. Given the variability of age definitions of AYAs in the literature, we did not set age cut-offs for inclusion; thus, a study was considered for inclusion if it reported including adolescents and/or young adults. The focus of the review is on issues facing AYAs at the time of diagnosis and treatment, thus papers solely describing the experiences of survivors were excluded. Prospective cohort studies covering post-treatment and studies including patients both on and off treatment were however considered for inclusion; thus, some of the studies included in our review included AYAs off treatment. We note that definitions of survivors also vary in the literature and we used the working definition adopted by the European Organisation of Research and Treatment for Cancer (EORTC) Survivorship Task Force of any person who has been diagnosed with cancer and is off treatment with curative-intent (with the exception of maintenance treatment) and disease free (has no evidence of active cancer) [20]. Individual case reports and abstracts from conference proceedings were also excluded. Duplicate records were removed.

### Evaluation and data extraction

HRQoL issues were extracted from primary sources and recorded using a data extraction form. This task was carried out for all eligible papers and shared between the reviewers (S.S., O.H., J.R., R.D.). The extraction forms were verified by A-S.D. who also addressed additional queries regarding eligibility of papers. A descriptive synthesis of the data was used because of the heterogeneity of studies in terms of research focus and methods of recording HRQoL outcomes. The data extraction sheet identifies the age range of participants, research objective, methodology used, and HRQoL issues assessed or described using formal measurement tools or captured using interviews. A separate data recording sheet was used for papers focusing on AYA measures with the intention to capture the HRQoL issues assessed by the measures.

## Results

### Literature search

**Fig. 1:**
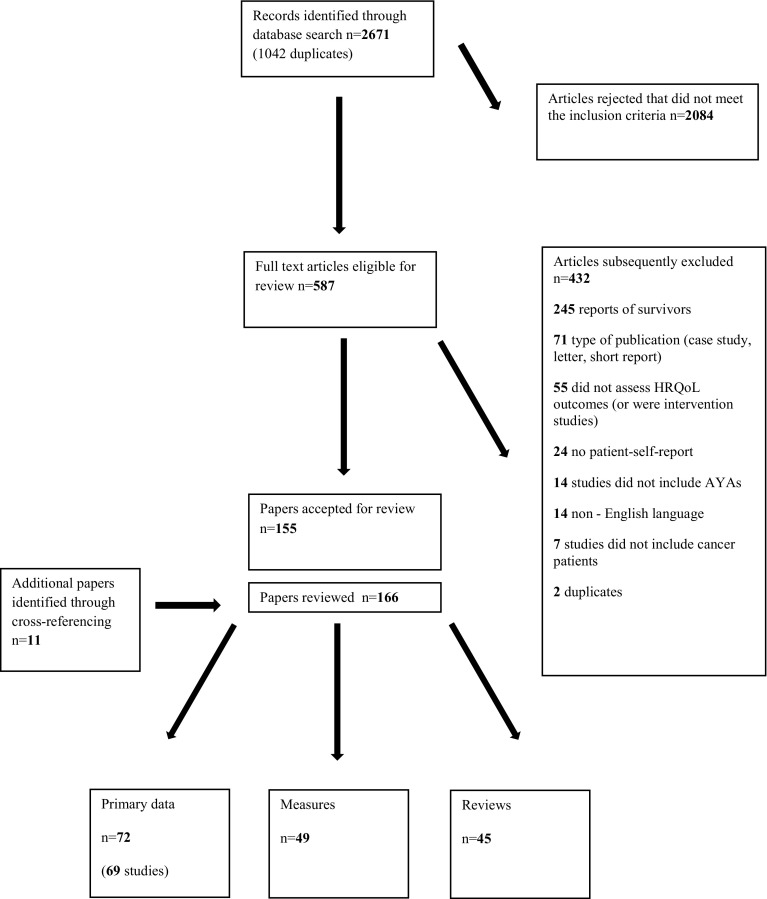
Flow chart of the paper selection process

The selection process generated 2671 hits (Fig. 1). Screening of titles and abstracts identified 587 (22%) papers for full review with agreement between reviewers for 1911 (72%) papers. The subsequent data extraction phase identified 432 papers of the 587 papers as ineligible for review with 245 papers describing the experiences of AYAs post-treatment and 187 rejected on the basis of subject matter (not reporting HRQoL issues), patient group (non-AYA with cancer), language (non-English) or type of publication (case study, conference report, proxy assessment). The additional 11 papers were identified through cross-referencing; thus, 166 papers were considered for review. There were 45 review papers, 49 papers describing the properties of questionnaires used with AYAs with cancer and 72 reporting the results of studies (69 in total) using HRQoL as an outcome assessment.

### Description of studies

Out of the 69 studies reviewed, 3 included only AYAs in their sample using the authors’ definition of AYAs [21–24] with an upper age of 39 years used as an inclusion criteria in the Adolescent and Young Adult Health Outcomes and Patient Experience (AYA HOPE) Study [23, 24]. Young adults were the focus of 6 studies [25–30] and again these were heterogeneous in terms of age range of inclusion with the lowest age of 20 used for 3 studies [26, 29, 30] and 47 years representing the oldest age [28]. Adolescents were described as the focus of 14 studies [31–44] with the age for inclusion ranging between 10 years [39, 40, 43] and 23 years [36]. The remaining studies reviewed included adolescents as part of a larger sample which also included children and 9 of these [45–53] treated adolescents (12/13 year olds) as a separate group.

Leukaemia, lymphoma, cancer of the central nervous system and sarcoma were amongst the most common disease groups. Several studies provided a comparison of HRQOL issues according to disease type (solid versus haematological cancers) [54] or subtype [acute myeloid leukemia (AML) and acute lymphoblastic leukemia (ALL)] [55], disease risk (Hodgkin lymphoma ‘high risk’ vs leukemia ‘low risk’) [56] and treatment status [57]. Some studies captured HRQoL across the disease/treatment trajectory looking at change over time [29]. Comparisons have been made with healthy individuals either in terms of reference to population norms [24, 57] or the inclusion of a separate sample of healthy participants [58]. Patients have also been compared according to age group (children versus adolescents) [45, 53, 55]. Finally, patients’ self-reports have been compared with proxy assessments in order to identify consistency in reporting of HRQoL issues [38].

The studies reviewed asked AYAs to describe or rate their HRQoL using interview questions or self-completed questionnaire assessments. Of the 69 studies reviewed, 11 [25, 28, 32, 34, 39, 42, 49, 50, 59–61] used interviews to evaluate the experiences of AYAs and capture HRQoL issues. Five studies [25, 34, 50, 59, 61] used open-ended, semi-structured interviews and 6 were more structured with a particular domain of interest including pain [32], fatigue [39, 49], body image [28], romantic relationships and fertility [42]. Three studies [32, 39, 60] used interviews to complement the completion of measures. The remaining 58 studies reviewed relied solely on the use of questionnaires which were either generic in their focus (PedsQL) [62] or measured a specific HRQoL concern (Reproductive Concerns Instrument) [38]. PROMS selected also varied according to their intended age group. Some studies asked AYAs with cancer to complete measures designed for adult respondents, for example, the EORTC core generic cancer quality of life questionnaire (EORTC-QLQ-C30) [63] was used in four of the studies reviewed [22, 26, 45, 55]. Indeed, Trevino et al. [30] noted that as psychological well-being and distress scales have not been validated with young adults, they had to look for measures validated in other populations (i.e. adults). The majority of studies we reviewed used measures which had been either specifically developed for AYAs, adolescents or young adults, or which had been adapted from paediatric or adult measures to capture the needs and concerns of AYAs.

### Description of measures

#### Adolescent and/or young adult-specific measures

Our review captured one measure which was specifically designed for both adolescents and young adults with cancer: The Cancer Needs Questionnaire-Young People (CNQ-YP) [64] which includes items relevant to young people aged 14–25 years. In addition, two other measures, one tumour-specific, the Dutch DUX questionnaire for lower extremity bone tumour patients (Bt-DUX) [65] and one generic, the Perceived Illness Experience (PIES) Scale [66], were developed using children as well as AYAs (up to 25 and 24 years respectively). We identified two adolescent measures which are generic in terms of their disease focus: The Adolescent Quality of Life Instrument [67] was piloted with 9–20 year olds and the Hopefulness Scale for Adolescents (HSA) [68]. One measure reviewed was designed for young adults with testicular cancer (aged 18–29 years): The Cancer Assessment for Young Adults—Testicular (CAYA-T) [69]. In addition, there was one measure designed for children and adolescents (8–18 year olds): The Quality of Life in Children and Adolescents with Cancer Scale (PEDQOL) [70]. The studies reviewed also used several generic measures developed for use with age groups covering adolescence, for example the Child Health Questionnaire (CHQ) [71] is relevant for children aged 10–15 years and the Behavioural Assessment System for Children (BASC) [72] is designed to be used with children aged 4–18 years. The DISAKIDS measure (DCGM-37) [73] is used with school aged children including adolescents [74]. The Activities Scale for Kids Performance Version (ASKp) [75] was designed for children aged 10–15 years and has since been used to assess physical function in adolescents with bone tumours aged 10–18.9 years [76] and the Pediatrics Outcomes Data Collection Instrument (PODCI) [77] provides an assessment of functional status in children and adolescents. The Pediatric Functional Assessment of Anorexia and Cachexia Therapy (peds-FAACT) [78] is specific to 7–17 years and includes additional peripheral items for 10–17 year olds. In addition, the PROMIS Pediatric Measures selected for use by Hinds et al. [79] were suitable for completion by 8–17 year olds.

#### Adapted existing measures

The majority of questionnaires captured in this review represent adaptations of paediatric or adult measures and thus include different age versions. The PedsQL [62] was the most common measurement scale of choice amongst the studies reviewed and used in 27 of the 69 studies The PedsQL measurement model captures both generic (core measure) and disease-specific aspects of measurement with the core instrument supplemented by disease-specific (e.g. cancer), tumour-specific (e.g. brain) or symptom-specific (e.g. fatigue) modules. In terms of age versions, the PedsQL offers an adolescent form for 13–18 years and was adapted by Ewing et al. [80] to create an AYA form appropriate for 16–24 years. The Pediatric Cancer Quality of Life Inventory (PCQL-32) [81] and the Quality of Life for Children with Cancer (QOLCC) [82] also have adolescent versions for 13–18 year olds. The Pediatric Advanced Care Quality of Life Scale (PAC-QoL) [83] has an adolescent version for 13–19 year olds. The KINDL measure of quality of life in chronically ill children has an oncology-specific as well as adolescent version (KINDL Kiddo) for children 13–16 years [84]. The Child Health and Illness Profile also has an adolescent edition (CHIP-AE) for 11–17 year olds [85]. There are also several measures developed with adults that have been adapted for use with younger respondents. The Health-related Hindrance Inventory (HRHI) [86] was adapted for use with adolescents and the Reproductive Concerns Instrument [87] was adapted for use with 12–18 year old females with cancer [38]. The Memorial Symptom Assessment Scale (MSAS 10–18) was also adapted for children and adolescents (aged 10–18 years) [88]. The Minneapolis–Manchester Quality of Life (MMQL) Questionnaire has an adolescent form (13–20 years) [89] and the Behavioural Affective and Somatic Experiences Scale has a child-form (BASES-C) [90] covering AYAs (up to 20 years). Finally, the 16-Dimensional Health-related Measure (16D) [91] represents an adaptation of the adult 15D measure and is suitable for 12–15 years.

### HRQoL issues

HRQoL issues extracted from interviews with AYAs or measurement concepts of instruments used in the studies reviewed are presented in Table 2.

**Table 2:** HRQoL issues generated from PROMs or interviews with AYAs

Measurement concept	Source	
	PROM^a^	Interview
Physical functioning		
*General* physical functioning/health status	Adolescent Quality of Life Instrument, ASKp, Bt-DUX, CHIP-AE, CHQ, CAYA-T, MMQL, KINDL Kiddo, PCQL-32, PEDQoL, PedsQL measures, PODCI, QOLCC, FACT-G, SF-36	[59]
*Symptoms* Pain/discomfort/hurt Fatigue, energy, loss of strength, insomnia, nausea, vomiting, diarrhoea, constipation, skin changes, dizziness, oedema, hair loss, mouth sores, weight loss, anorexia, cachexia, appetite, desire to eat	CHQ, CHIP-AE, CNQ-YP, HRHI, MSAS 10–18, PAC-QoL, PCQL-32, PedsFAACT, PedsQL measures, PODCI, PROMIS, QOLCC, 16-D, HUI, EORTC QLQ-C30, SCNS, PGWB	[25, 28, 32, 34, 39, 42, 49, 50, 59, 61]
*Physical limitations*	CNQ-YP, DISAKIDS, PedsQL, PODCI, PROMIS, 16-D, EORTC QLQ-C30	
Cognitive functioning		
*Concentration, memory*	CAYA-T, MMQL, MSAS 10–18, PedsQL, PEDQOL, PCQL-32, QOLCC, HUI, EORTC QLQ-C30	[49]
Activity limitation		
* Self-care, education, work, sport/leisure/hobbies, social/family activities*	Adolescent Quality of Life Instrument, BASES-C, CAYA-T, CHQ, CNQ-YP, PedsQL, PIES, 16-D, HUI, EORTC QLQ-C30, SCNS	[25, 34, 39, 49, 50, 59–61]
* Loss of “normal life”*		[50, 60, 61]
* Plans for the future*	CNQ-YP	[49]
*Abilities*/realisation of talents/difficulty competing	CHIP-AE, CNQ-YP	[49]
*Positive effects* of restricted activities (education)		[61]
Relationships with others		
*General* social functioning	BASES-C, Bt-DUX, CAYA-T, DISAKIDS, MMQL, PAC-QoL, PCQL-32, QOLCC, EORTC QLQ-C30, FACT-G, SF-36	[60]
*Friends* Loss of friendships, disconnected, isolated from others, peer rejection, bullying	CNQ-YP, DISAKIDS, KINDL Kiddo, PEDQOL, PedsQL, PIES, PROMIS, 16-D	[25, 34, 50, 59, 61]
*Family* Strained relationships Dependency on others/lack of autonomy	Adolescent Quality of Life Instrument, CHQ CNQ-YP, DISAKIDS, KINDL Kiddo, PEDQOL	[25, 34, 49, 50, 59]
*Communication*/refusal to talk	PedsQL, QOLCC, 16-D	[34, 49]
*Romantic*/*sexual* relationships	CAYA-T, CNQ-YP, MMQL, SCNS, IOC	[42]
Prospects for *future relationships*		[42]
*Impact on others* Emotional, time impact, parental behaviour, over-protectiveness	CHQ, CNQ-YP, PIES, SCNS	[34, 61]
*Positive effects* Greater value placed on friendships, appreciation of the support from others, strengthened family ties, opportunities for new friendships, positive attention from others		[25, 34, 42, 50]
Emotional functioning		
*Frustration, anger, upset, anxiety, depression, fear, vulnerability, preoccupation with illness, meaning of being ill, boredom*	Adolescent Quality of Life Questionnaire, BASC, BASES-C, Bt-DUX, CAYA-T, CHQ, CNQ-YP, DISAKIDS, KINDL Kiddo, MMQL, MSAS 10–18, PAC-QoL, PCQL-32, PEDQOL, PROMIS, PIES, QOLCC, 16-D, McGill Quality of Life Questionnaire, MASC, HUI, EORTC QLQ-C30, PGWB, HADS, Beck Depression Inventory, STAI-S, FACT-G, SF-36	[25, 34, 49, 50, 59–61]
*Loss of confidence/self-esteem*	CHQ, PODCI	[34, 42, 49]
*Lack of motivation*		[49]
*Hope*	HSA	[34]
*Improved self-appraisals* (maturity, better person), self-esteem		[34, 50]
Fertility		
	Reproductive Concerns Instrument, HUI	[42]
Body image		
	CNQ-YP, Bt-DUX, MMQL, MSAS 10–18, PEDQOL, PedsQL, PIES, and 16-D	[28, 34, 42]
Spiritual wellbeing/outlook on life		
*Spiritual*	CAYA-T, McGill Quality of Life Questionnaire	[60]
*Outlook on life* *Positive attitude to life*	MMQL, PWBS	[25, 50]
Financial difficulties		
	EORTC QLQ-C30, SCNS	

^a^PROMS used in the studies reviewed

Adolescent and/or Young Adult PROMs: Cancer Needs Questionnaire-Young People (CNQ-YP) [64], Bt-DUX [65], Perceived Illness Experience (PIES) [66], Adolescent Quality of Life Instrument [67], Hopefulness Scale for Adolescents (HSA) [68], Cancer Assessment for Young Adults—Testicular (CAYA-T) [69], Quality of Life in children and adolescents with cancer PEDQOL [70], Child Health Questionnaire (CHQ) CF 871 [71], The Behavioral Assessment System for Children (BASC) [72], DISAKIDS (DCGM-37) [73], Activities Scale for Kids, performance version (ASKp) [75], Pediatrics Outcomes Data Collection Instrument (PODCI) [77], Pediatric Functional Assessment of Anorexia and Cachexia Therapy (peds-FAACT) [78], PROMIS Pediatric Measures [79], MASC Multidimensional Anxiety Scale for Children [99]

Adapted existing measures: *Paediatric* Pediatric Quality of Life Inventory (PedsQL) Core [62, 80], Pediatric Cancer Quality of Life Inventory (PCQL-32) Varni et al. [81], Quality of Life for Children with Cancer (QOLCC) [82], The Pediatric Advanced Care Quality of Life Scale (PAC-QoL) [83], KINDL Kiddo [84], Child Health and Illness Profile (CHIP-AE) [85]. *Adult* Health-related Hindrance Inventory (HRHI) [86], Reproductive Concerns Instrument [87], Memorial Symptom Assessment Scale (MSAS 10–18) [88], Minneapolis-Manchester Quality of Life (MMQL) Questionnaire [89], Behavioural Affective and Somatic Experiences Scale (BASES-C) [90], 16-Dimensional Health-related Measure (16-D) [91]

Non-AYA specific: EORTC QLQ-C30 [63], Supportive Care Needs Survey (SCNS) [94], Impact of Cancer Scale (IOC) [95]. *Note* Roper used two questions from the IOC social and relationship scale, Health Utilities Index (HUI) [96], Psychological Wellbeing Scale (PWBS) [98]. Monteiro et al. [26] used only the personal growth subscale of the PWBS, Psychological General Wellbeing Index (PGWB) [100], McGill Quality of Life Questionnaire [101] Trevino et al. [30] used only the psychological well-being and existential subscales, Beck Depression Inventory [102], State Trait Anxiety Scale (STAI) [103], Hospital Anxiety and Depression Scale (HADS) [104], Functional Assessment of Cancer Treatment (FACT-G) [105], The MOS 36-Item Short-Form Health Survey (SF-36) [106]

#### Physical

AYAs talked about their symptoms such as fatigue, loss of strength, pain, cognitive difficulties, hair loss, impaired appetite and desire to eat [28, 32, 34, 39, 42, 49, 50, 59, 61]. Symptoms were recognised as a major theme in Moody et al.’s exploratory study [61] and as a HRQoL domain by Hinds et al. [59]. Physical functioning of AYAs with cancer including health status, mobility and symptoms (disease- and treatment-related) such as pain, fatigue, nausea, discomfort, difficulty sleeping, mobility, anorexia and cachexia, is assessed by a number of PROMs (PedsQL instruments, CHQ, CHIP-AE, Bt-DUX, Adolescent Quality of Life Instrument, CAYA-T, DCGM-37, ASKp, PODCI, PROMIS measures, PCQL-32, QOLCC, PACQOL, BASES-C, MMQI, HRHI, KIDDO and 16-D). The PedsQL also includes a fatigue-specific module. Five studies recorded symptom prevalence and impact using symptom distress or checklist measures [27, 36, 92, 93].

#### Cognitive functioning

Difficulties thinking and concentrating were described by adolescents in Chiang et al.’s study [49] as impacting on their school attendance. Cognitive functioning in terms of attentiveness, memory and cognitive fatigue also forms part of the CAYA-T, PedsQL (within the school functioning subscale and the PedsQL Fatigue measure).

#### Restricted activities

Symptoms such as fatigue, lack of strength and motivation as well as time spent undergoing medical treatment compromised participants’ ability to engage in everyday activities, partake in sports, attend school and interact with others [25, 34, 39, 49, 50, 59, 60]. When discussing the impact of illness and treatment, adolescents often made reference to milestones their peers had reached such as selecting colleges and learning to drive [60]. Parents’ over-protectiveness was also perceived by adolescents as a barrier to engaging in everyday activities [49]. For adolescents in Chiang et al.’s study [49], this lack of participation in activities was expressed as impairing their self-performance and compromising the realisation of talents which also impacted on future life plans. Participants of Moody et al.’s [61] study described feeling trapped and bored by their lack of freedom and loss of a normal life. Poor attendance at school resulted in adolescents falling behind their peers [49, 61]. However, for participants of Momani et al.’s study [50], missing school was described as a positive effect of being ill.

PROMs also evaluate the impact of physical health status and cognitive functioning on the ability to carry out daily activities, most notably self-care, work, education and hobbies. (CNQ-YP, PIES, Adolescent Quality of Life Instrument, CAYA-T, CHQ, PedsQL, 16-D). The PedsQL core measure assesses aspects of school functioning with the AYA form adapted to read study/work. In addition, the CHIP-AE provides an assessment of achievement (academic and work).

#### Relationships with others

Restricted social interactions were a recurring theme leaving AYAs feeling disconnected and isolated from their peers [25, 34, 50, 59, 61]. There were reports of friends “keeping their distance” [34] as well as an insight into “true friends” [34, 61]. In addition to losing friendships, there were accounts of opportunities to establish new friends amongst fellow patients [50]. Participants also described greater value placed on relationships with others with friendships taking on a new meaning and family ties strengthened and these were seen as an important source of comfort [25, 34, 50].

Increased dependency on others came at a time when adolescents had recently gained independence and resulted in feelings of loss of control [25]. AYAs in Enskär’s et al.’s study [34] also talked about strained relationships with family members.

Opportunities for romantic relationships were also described as limited and took on a lower priority due to symptoms of fatigue and nausea as well as lowered self-esteem and prolonged hospital stays [42]. In addition, anxiety surrounding fertility was also reported [41] and led to concerns about prospects for future relationships. Stinson et al. [42] also explored the impact of cancer on adolescents’ sexual relationships and discovered that, in contrast to the assumptions held by parents, AYAs perceived little impact.

Social functioning is covered by most of the measures used in the studies reviewed (CNQ-YP, Bt-DUX, CAYA-T, PCQL-32, QOLCC, PedsQL, PIES, CHQ, DCGM, Adolescent Quality of Life Instrument, PROMIS Measures, PAC-QoL, KINDL/KIDDO, BASES-C, MMQL and 16-D). The PEDQOL and KINDL Kiddo distinguishes between relationships with family and friends. Social exclusion including peer rejection and bullying is assessed by the DCGM, PedsQL and PIES. In addition to measuring the impact on family relationships and interactions, some measures examine the perceived impact on family members, for example, emotional/time pressures on parents, limited family activities and family cohesion are assessed by the CHQ while parental behaviour represents a domain of the PIES. Emotional reactions of others also formed part of Enskär et al.’s problem list [34] and concern over the feelings of family members was voiced in Moody et al.’s study [61]. Dyson et al. [21] used The Supportive Care Needs Survey (SCNS) [94] to assess AYAs’ concerns about worries of those close to them.

Intimate relationships are a domain of the MMQL and sexual functioning is assessed as part of the CAYA-T and formed part of the supportive needs assessment used by Dyson et al. [21]. Roper et al. [27] used the Impact of Cancer scale [95] to assess intimate relationships in young adults.

#### Fertility

Concerns over fertility are the focus of one of the measures reviewed (Reproductive Concerns Instrument, used by Quinn et al. [38]) and forms part of the Health Utilities Index [96] used by Yaris et al. [97]. Trevino et al. [30] discussed loss of fertility in the context of causing significant unique cancer-related grief amongst young adults.

#### Emotions

Interviews with AYAs provided accounts of their emotional reactions to being ill. Mood was identified as a HRQoL domain by Hinds [59] and emotional reactions were a major theme reported by Moody et al. [61]. Participants described a greater tendency to become upset [50], as well as feelings of vulnerability [25], frustration [49], anger [61], and fear over mortality [61]. The impact of cancer on self-confidence [34] and self-esteem was also reported [34, 42] although improvements to self-appraisals were also described such as becoming a better, more positive and less selfish person [34, 50] as well as feelings of greater maturity [34].

Feelings are evaluated alongside relationships in the (CNQ-YP). Assessments of emotional or psychological functioning amongst AYAs with cancer include mood (Adolescent Quality of Life Questionnaire, BASES-C), anxiety (BASC, PROMIS, PedsQL), depression (BASC, PROMIS), sadness (PedsQL) preoccupation with cancer (PIES), concerns for the future (PedsQL, SCNS), fear of the unknown (PedsQL), lack of motivation, anger (PROMIS, PedsQL), irritability, vulnerability, hopefulness (HSA), confidence and self-esteem/self-worth (CHQ, PODCI).

#### Body image

Emotions relating to appearance issues such as hair loss formed part of the problem list discussed by AYAs in Enskär et al.’s study [34] and was the focus of Snöbohm et al.’s study [28]. Adolescents in Stinson et al.’s study described feeling less attractive and desirable to others which in turn impacted on their self-esteem and confidence in engaging in romantic relationships [42]. Physical appearance or perceived body image is evaluated as part of the PIES, PEDQOL, Bt-DUX, MMQL, 16-D and PedsQL Cancer module which includes the item “I don’t look like myself”.

#### Spirituality and outlook on life

The impact of illness on spirituality was explored amongst children and adolescents of Kamper et al.’s study [60] and altered, often positive, perspectives on life were described by adolescents [50] and young adults [25]. The Adolescent Quality of Life Instrument evaluates the meaning of being ill. Spirituality is covered by the CAYA-T and is the focus of the Spiritual Quality of Life Questionnaire used in Kamper et al.’s interviews [60]. Personal growth amongst young adults with cancer was evaluated by Monteiro et al. [26] by using the Personal growth subscale of the Psychological Well-Being Scale [98]. The MMQL assessment also provides an insight into outlook on life.

#### Financial difficulties

Zareifar et al. [55] used the EORTC QLQ C30 [63] to provide an evaluation of financial difficulties. Roper et al. [27] also measured the use of supportive care services and discovered that financial concerns were identified as a major reason for accessing such services.

## Discussion

From our review of studies reporting the HRQoL impact of cancer on AYAs and our examination of the content of AYA-specific or adapted PROMs, we have generated a comprehensive list of HRQoL issues relevant to AYAs with cancer. Researchers interested in measuring the impact of cancer and its treatment on the HRQoL of AYAs have used a wide range of validated instruments. Measures developed with and for adults, such as the EORTC QLQ-C30 provide opportunities for the measurement of HRQoL concepts, in particular symptoms such as pain, fatigue, nausea and vomiting that do not begin and end in the AYA years. Adult and paediatric measures have also been adapted to the specific issues relevant to AYAs and have AYA versions (PedsQL [62]) and finally there are measures which have been developed with and designed specifically for AYAs (CNQ-YP) [64]. Choice of measurement tool is driven by the purpose of the study with the focus of some studies refined to a particular aspect of HRQoL. The types of HRQoL issues captured from these studies thus reflect the purpose of the study and measure used.

Interviews, as an alternative or addition to the completion of questionnaires, provide AYAs the opportunity to evaluate aspects of HRQoL which might not be covered in existing measures. In addition, interviews with AYAs have the potential of offering greater insight into the HRQoL issues as expressed by AYAs with issues captured often incidental to the main purpose of the research, for example, Kamper et al.’s [60] interviews on spiritual wellbeing also generated accounts of emotional and social functioning.

There is widespread agreement within the literature that adolescence and early adulthood mark a period of great turmoil with accelerated physical, cognitive and emotional growth and with this comes challenges regarding sense of self and autonomy, interpersonal relationships (family, romantic and friends) and decision making regarding the future [107]. Indeed, it has been argued that adolescents and young adults are not a “homogeneous entity” [108] thus they might differ from each other in terms of HRQoL concerns—for adolescents, independence from parents, peer relationships and educational achievement might be more pertinent while young adults might be more concerned with career choices, financial independence and establishing intimate relationships in view of starting a family. The challenges presented during this period of significant developmental transition are exacerbated by a diagnosis of cancer. Thus, HRQoL measurement during this developmental phase needs to be sensitive to these unique challenges.

The studies and measures reviewed in the current paper highlight the wide spectrum of HRQoL issues facing AYAs and include physical, cognitive, emotional, social and spiritual functioning which fit with the general, i.e. non-age specific definition of HRQoL offered by the World Health Organization. Within these HRQoL domains, there are issues which are particularly relevant to AYAs which may not be so familiar or relevant to children or older patients, for example, within the generic core PedsQL, social functioning includes relationships with peers, social exclusion and bullying with the AYA version including items relating to study and work. Additional domains covered in this review include inability to engage in activities enjoyed by peers, body image, concerns over reproductive capacity and intimate relationships. In addition, this review revealed that not all consequences of a cancer diagnosis and treatment are viewed by AYAs as negative with descriptions of greater maturity, becoming a better person, positive effects of missing school, increased family time and improved relationships with others reported (e.g. [34, 50]).

## Limitations

One of the main limitations of this review is that the HRQoL concerns of AYAs presented in this paper are confined to the content of questions asked of patients. With a limited number of interview-based studies giving AYAs the opportunity to rate aspects of their cancer or treatment not covered by the questionnaires, it is likely that this review might have missed important issues. The studies reviewed were mostly cross-sectional and included only small numbers of patients; thus, caution is required before making generalisations about the HRQoL concerns of this patient group. In addition, formal comparisons with other age groups and between genders fell beyond the scope of this review.

This review is also limited in terms of its descriptive synthesis of the data. The heterogeneous nature of the studies reviewed in terms of their focus, outcomes assessed and measures used resulted in data being presented in different formats; comparisons between studies were difficult and we were not in a position to present prevalence figures for individual HRQoL issues. While our review captured issues from measures used with AYAs in the studies reviewed, it is possible that we overlooked some adult measures with content relevant to AYAs. In addition, age definitions of adolescents and young adults varied across studies and the number of studies focusing exclusively on adolescents and young adults was limited, which meant we relied on findings from studies including AYAs as part of a sample including children or older adults. Care was taken to report only issues relating to AYAs although this was compromised in studies not providing a separate treatment of the responses of AYAs. Thus, the list of issues might not necessarily be specific to AYAs. In addition, it could be argued that we should not treat AYAs as one group [108]. However, our review was not designed to produce separate lists according to age and we did not attempt to draw age comparisons. Although we were interested in issues facing AYAs at diagnosis and treatment, our review included studies with AYAs off treatment as part of their sample; thus, our list of issues might include those relevant to patients post-treatment. However, it was not the intention of this review, to make claims regarding issues which are likely to remain post-treatment and the experience of AYA cancer survivors.

Further avenues for research could include a more detailed analysis of the prevalence of the HRQoL issues amongst AYAs with cancer using a more conservative age definition such as 14–25 years. In addition, comparisons with the cancer experiences of other age groups would also merit further study in order to help identify and address the unique needs of AYAs.

## Conclusion

To the best of our knowledge, this review represents the first attempt to systematically review studies and the PROMS they have used to capture HRQoL issues as described by AYAs undergoing treatment for cancer. From this review, we have provided a comprehensive list of HRQoL issues for this age group which might be of value to clinicians in providing insight into the complexities of the cancer experience for AYAs which in turn will help support their consultations with AYAs. This review is also informative to AYAs themselves and their significant others in terms of preparing them for what life might be like as an AYA with cancer.
